# Distinct association patterns of chemokine profile and cardiometabolic status in children and adolescents with type 1 diabetes and obesity

**DOI:** 10.3389/fendo.2024.1335371

**Published:** 2024-07-23

**Authors:** Anita Špehar Uroić, Maša Filipović, Alan Šućur, Tomislav Kelava, Nataša Kovačić, Danka Grčević

**Affiliations:** ^1^ Division of Pediatric Endocrinology and Diabetes, Department of Pediatrics, University Hospital Center Zagreb, Zagreb, Croatia; ^2^ Pediatric Clinic, Children’s Hospital Zagreb, Zagreb, Croatia; ^3^ Department of Physiology and Immunology, School of Medicine, University of Zagreb, Zagreb, Croatia; ^4^ Laboratory for Molecular Immunology, Croatian Institute for Brain Research, School of Medicine, University of Zagreb, Zagreb, Croatia; ^5^ Biomedical Research Center Šalata (BIMIS), School of Medicine, University of Zagreb, Zagreb, Croatia; ^6^ Department of Anatomy, School of Medicine, University of Zagreb, Zagreb, Croatia

**Keywords:** children, type 1 diabetes mellitus, obesity, chemokines, chemokine receptors

## Abstract

**Objective:**

We compared peripheral blood (PBL) chemokine ligand/receptor profiles in children and adolescents with type 1 diabetes mellitus (T1D) or obesity (OB) (both involving inflammation and vascular complications) to identify their associations with cardiometabolic risk factors.

**Materials and methods:**

PBL samples from children and adolescents (12–18 years) included: healthy controls (n=29), patients with T1D (n=31) and OB subjects (n=34). Frequency of mononuclear cell populations and chemokine receptor expression (CCR2, CCR4, CXCR3, CXCR4) were determined by flow cytometry. Chemokine levels of CCL2, CCL5, CXCL10 and CXCL11 were measured by bead-based assay and CXCL12 by ELISA. Data were correlated with cardiovascular, metabolic and inflammatory parameters.

**Results:**

The proportion of CD14^+^ monocytes was higher in T1D, whereas the proportion of CD19^+^ B lymphocytes was higher and CD3^+^ T lymphocytes was lower in OB. The level of CCL2 was higher in T1D (241.0 (IQR 189.6–295.3) pg/mL in T1D vs 191.5 (IQR 158.0–254.7) pg/mL in control, p=0.033), CXCL11 was lower in OB (6.6 (IQR 4.9–7.7) pg/mL in OB vs 8.2 (IQR 6.9–11.3) pg/mL in control, p=0.018) and CXCL12 was lower in both diseases (2.0 (IQR 1.8–2.5) ng/mL in T1D, 2.1 (IQR 1.9–2.4) ng/mL in OB vs 2.4 (IQR 2.2–2.5) ng/mL in control, p=0.016). Numerous significant associations were found for chemokine ligand/receptor profiles and clinical data. Among these, we are suggesting the most important indicators of cardiometabolic risk in T1D: positive associations of CCR2^+^ monocytes with blood pressure and CCL12 levels with urine albumin-to-creatinine ratio (ACR), inverse association of CXCR3^+^ B lymphocytes with AST but positive with triglycerides; and OB: positive associations of CXCL12 levels with triglycerides and AST/ALT, inverse association of CCR4^+^ and CXCR3^+^ monocytes with ACR. Both diseases share positive associations for CCR4^+^ T lymphocytes and blood pressure, inverse associations of CXCR4^+^ subsets with ACR and CXCR3^+^ T lymphocytes with lipid profile.

**Conclusion:**

Significantly changed chemokine ligand/receptor profiles were found in both T1D and OB even at a young age. Although different associations with cardiometabolic risk factors indicate disease-specific changes, overlapping pattern was found for the associations between CCR4^+^ T lymphocytes and vascular inflammation, CXCR4^+^ subsets and albuminuria as well as CXCR3^+^ T lymphocytes and dyslipidemia. Thus, chemokine axes might present potential therapeutic targets for disease-related morbidity.

## 1 Introduction

A number of chronic conditions leading to serious metabolic disturbances are often underlined by low-grade persistent inflammation (1, 2). Therefore, targeting inflammatory mediators in addition to disease-specific therapy may provide significant benefits for pediatric patients with different autoimmune, cardiovascular and metabolic disorders. Among others, chemokines, released by the local endothelium, resident and invading immune cells, are crucial for leukocyte infiltration into target tissues (3, 4).

Type 1 diabetes mellitus (T1D) is one of the most prevalent chronic autoimmune diseases in children (5). It is characterized by insulin deficiency due to destruction of insulin-producing β-cells and resulting disturbances of carbohydrate, protein and lipid metabolism (6–8). It is estimated that more than a million children (<20 years) are living with T1D worldwide, with almost a third coming from Europe (5). None of the currently available therapies can achieve ideal glycemic control and absolutely prevent long-term vascular damage, necessitating additional insights into diabetes pathophysiology and novel treatment options (3).

Childhood obesity (OB) has emerged as an important public health problem worldwide, affecting >30% of school-age children in the USA and Europe (9–11). The most common cause of pediatric OB is a positive energy balance combined with a genetic predisposition for weight gain. Its rising prevalence is linked to serious comorbidities, including T2D, hypertension, metabolic dysfunction-associated fatty liver disease (MAFLD), obstructive sleep apnea and dyslipidemia, which increase the risk of cardiovascular diseases (9, 12, 13). The adolescent OB population additionally displays reproductive dysfunction and psychosocial problems (9, 13, 14).

In both diseases, serious macrovascular and microvascular complications may result from endothelial damage driven by inflammation and metabolic dysfunction (15). While in OB children, lipid overload plays a major role in the induction of inflammation (16), in T1D, inflammation is triggered by the effects of advanced glycation end-products and by the activation of Toll-like receptors, both of which initiate inflammatory signaling cascades (17, 18).

The role of chemokine activity in the course of T1D and OB has recently attracted great interest (18–24). Chemokines are ubiquitously produced in response to microbial products or tissue damage, aiming to recruit cells expressing chemokine receptors. In addition to chemoattraction, chemokines display activities influencing angiogenesis, vascular endothelium activation, production of vasoactive mediators and cytokines as well as modulation of leukocyte functional properties. Chemokines thus have a crucial role in inducing inflammatory processes and mediating vascular damage, emerging as potential therapeutic targets (20, 25, 26). Elevated levels of CXCL10 and CXCL11, and concomitant lower lymphocyte expression of the corresponding receptor CXCR3 were found in T1D, suggesting a role in endothelium remodeling (3, 27). Chemokines CXCL10 and CXCL11 may also contribute to inflammation, insulin resistance and atherosclerosis in OB (4, 28, 29). The angiogenic role of CXCL12 could be detrimental in the context of diabetic nephropathy and retinopathy (30). On the other hand, reports on CXCL12/CXCR4 signaling in cardiovascular disease associated with OB suggest a protective role (20). Numerous studies have investigated the role of CCL2 and CCL5 in the development of metabolic complications and nephropathy in T1D (31–33). Research demonstrated the association of CCL5 with metabolic and regulatory mechanisms in OB, such as central energy control, food intake and hypothalamic temperature regulation (4, 34).

As T1D and OB both include cardiometabolic abnormalities that have been driven at least in part by underlying inflammatory processes, the first objective of the study intended to compare peripheral blood (PBL) samples from T1D and OB patients with the matched healthy participants to confirm that cardiometabolic and inflammatory parameters are changed with the disease, in parallel with the immune cell subsets and chemokine levels. Chemokines orchestrate the migration of immune cells expressing corresponding chemokine receptors; therefore, the second objective was to determine disease-specific changes in chemokine receptor profile on major immune cell subsets compared to control. Final objective aimed to assess within-disease association of chemokine ligands/receptors and cardiometabolic risk factors in order to identify potential markers of cardiometabolic complications, as indicators for early intervention as well as future therapeutic targets.

## 2 Materials and methods

### 2.1 Patients

The study included 94 children and adolescents (12–18 years) recruited from the outpatient clinic or inpatient settings of the Department of Pediatrics, University Hospital Center (UHC), Zagreb. The study was approved by the ethics committees of the School of Medicine, University of Zagreb (641–01/18–02/01) and UHC Zagreb (02/21 AG). All patients and their parents provided written informed consent for participation in the study. All procedures were carried out according to the Declaration of Helsinki.

T1D was diagnosed using the current criteria of the International Society for Pediatric and Adolescent Diabetes (ISPAD) (35). OB was defined according to body mass index (BMI) >95th percentile (36), calculated and presented as BMI Z score using the growth charts by the US Centers for Disease Control and Prevention (37). Subjects with any acute disease, other chronic illness or immunomodulatory therapy were excluded. Participants were divided into three groups: patients with T1D for ≥5 years following diagnosis, OB patients with BMI >95th percentile (BMI Z score >1.64 SD) and control healthy children of matching age and sex (**Supplementary Table 1**).

The following demographic, clinical and laboratory data were collected: age, sex, BMI Z score, diastolic/systolic blood pressure (DBP/SBP), C-reactive protein (CRP), fibrinogen (FIB), lipid profile (total cholesterol (TC), LDL-C, HDL-C, triglycerides (TG)), alanine and aspartate transaminases (ALT, AST), urine albumin-to-creatinine ratio in two consecutive first morning samples (ACR) for all participants; T1D duration (T1DD), HbA1c, and insulin dose (ID) for T1D patients; fasting blood glucose (FBG), blood insulin (INS), and homeostatic model assessment for insulin resistance (HOMA-IR) for OB patients (**Supplementary Table 1**).

### 2.2 Isolation of plasma and peripheral blood mononuclear cells

PBL samples (3–4 mL) were obtained between 9:30 and 11:30 AM during routine clinical assessment in EDTA-coated tubes. PBL mononuclear cells (PBMCs) and plasma were separated using Histopaque (Sigma−Aldrich, Saint Louis, MO, USA). Plasma samples were stored at -20°C for further analysis, whereas PBMCs were immediately used for immunophenotyping.

### 2.3 Immunophenotyping of peripheral blood cells

Immunophenotyping of PBMCs (T lymphocytes, B lymphocytes, and monocytes) for chemokine receptor expression was performed by an Attune flow cytometer (Thermo Fisher Scientific, Waltham, MA, USA) using commercially available monoclonal antibodies against specific cell markers (anti-CD3 for T lymphocytes; anti-CD19 for B lymphocytes; anti-CD14 for monocytes; anti-CCR2, anti-CCR4, anti-CXCR3 and anti-CXCR4 for chemokine receptors) (details of monoclonal antibodies used for flow cytometry analysis are shown in **Supplementary Table 2**). Analysis was performed based on gates defined according to unlabeled cells or “fluorescence minus one” controls by FlowJo software (FlowJo, v10, Ashland, OR, USA).

### 2.4 Cytometric bead-based immunoassay

Plasma levels of CCL2, CCL5, CXCL10 and CXCL11 were determined using the LEGENDplex Human Proinflammatory Chemokine Mix and Match Subpanel cytometric bead-based assay (BioLegend, San Diego, CA, USA) according to the manufacturer’s instructions, using a BD FACSAria IIu flow cytometer (BD Biosciences, San Jose, CA, USA). Briefly, plasma samples were incubated with beads coated with specific capture antibodies, which were identified by their scatter properties and APC fluorescence intensity. Samples were then washed and incubated with secondary antibodies conjugated with biotin and detected by streptavidin-PE. The concentration of the bound analyte was determined by the intensity of PE fluorescence using the provided standards and LEGENDplex Data Analysis Software (BioLegend).

### 2.5 Enzyme-linked immunosorbent assay

Plasma levels of CXCL12 were determined by enzyme-linked immunosorbent assay (ELISA) (Human CXCL12/SDF-1a, Quantikine Immunoassay; R&D Systems, Minneapolis, MN, USA) according to the manufacturer's instructions. Briefly, samples were incubated on pre-coated plates, washed and incubated with horseradish peroxidase-conjugated specific antibodies. Reactions were visualized with a substrate solution (tetramethylbenzidine) and arrested with sulfuric acid. Optical density was determined on a microplate reader set to 450 nm (GloMax Explorer Multimode Microplate Reader, Promega, Madison, WI, USA).

### 2.6 Statistical analysis

Most variables did not follow a normal distribution (determined using the Kolmogorov−Smirnov test for normality); therefore, data are presented as the median and interquartile range (IQR). Group differences were assessed by the Mann−Whitney test or Kruskal−Wallis with Conover *post-hoc* test using MedCalc (version 19.1.6; MedCalc Software Ltd., Ostend, Belgium). Associations between variables were assessed by the Spearman’s rank correlation using SPSS (version 26; IBM Corp, Armonk, NY, USA). Statistical significance was set to *α* < 0.05.

## 3 Results

### 3.1 Metabolic, cardiovascular, and inflammatory parameters in T1D and OB

**Figure 1:**
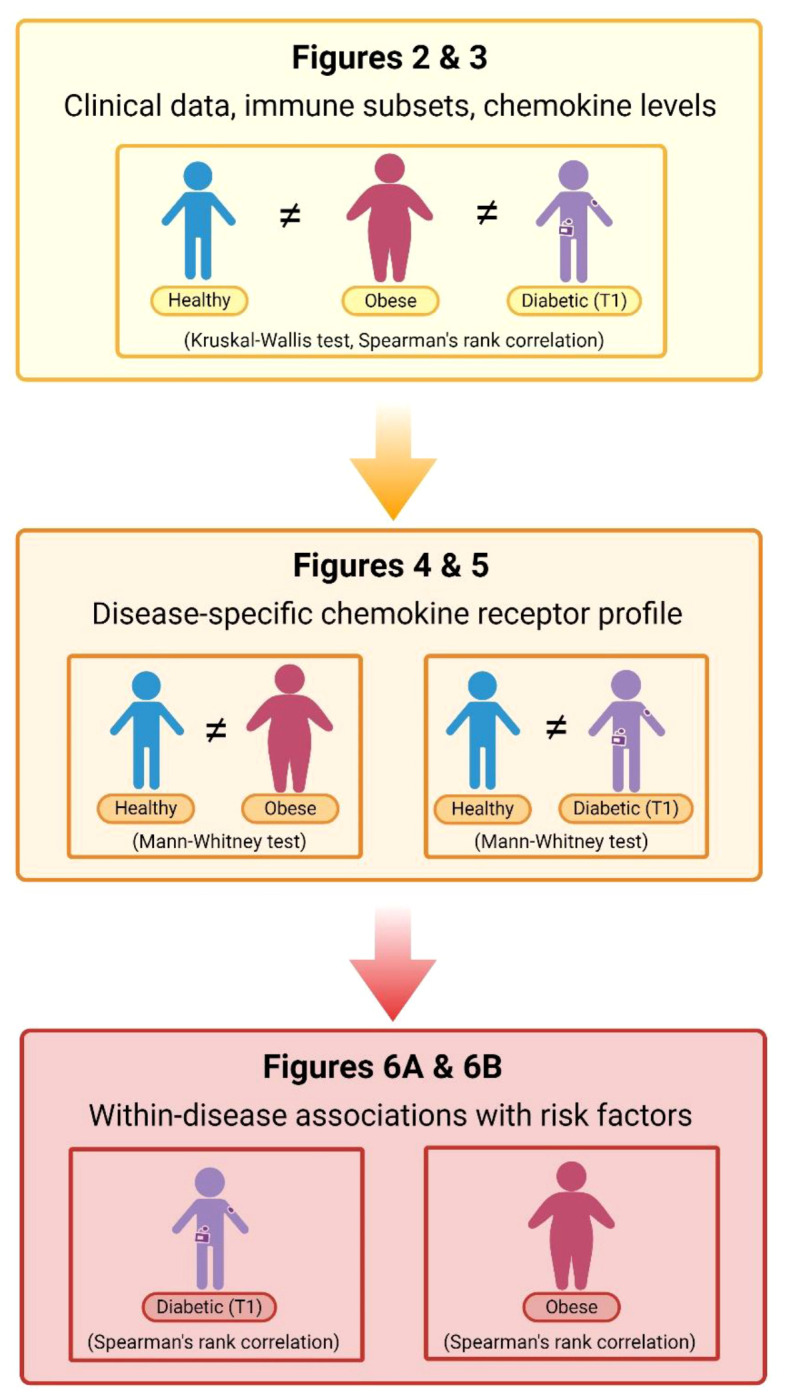
Flow–chart of data analysis. Data were analyzed in three steps: evaluation of clinical data (cardiometabolic and inflammatory parameters) in parallel with the immune cell subsets and chemokine levels in peripheral blood (PBL) samples from patients with diabetes mellitus type 1 (T1) and obesity compared to the matched healthy participants (using Kruskal–Wallis following by Conover *post-hoc* test for further group-to-group comparisons or Spearman’s rank correlation); profiling of disease-specific chemokine receptor expression by comparing healthy participants and individual disease group (using Mann–Whitney test); and, assessment of within-disease associations between chemokine ligands/receptors and cardiometabolic risk factors for each disease separately (using Spearman’s rank correlation). Corresponding figures are noted on the chart. Created with BioRender.com.

The study groups of children and adolescents were characterized as follows: nonobese T1D participants (n=31) had HbA1c 8.2 (7.4–9.2)% (equal to 66 (57–77) mmol/mol), T1DD 10.0 (6.6–11.9) years, ID 0.80 (0.67–0.98) U/kg and BMI Z score -0.12 (-0.69–0.68) SD; OB participants (n=34) had FBG 4.7 (4.4–5.0) mmol/L, blood INS 19.7 (14.9–27.4) μIU/mL, HOMA-IR 4.3 (3.0–6.2) and BMI Z score 2.4 (2.2–2.5) SD. The control group (n=29) included sex- and age-matched healthy participants with no history of T1D, OB, inflammatory, autoimmune or metabolic disorders. Data were analyzed in three steps: evaluation of clinical data and immune status in all three groups; profiling of disease-specific chemokine receptor expression by comparing individual disease with control; assessment of within-disease associations between chemokine ligands/receptors and cardiometabolic risk factors for each disease separately (T1D and OB) (**Figure 1**).

**Figure 2:**
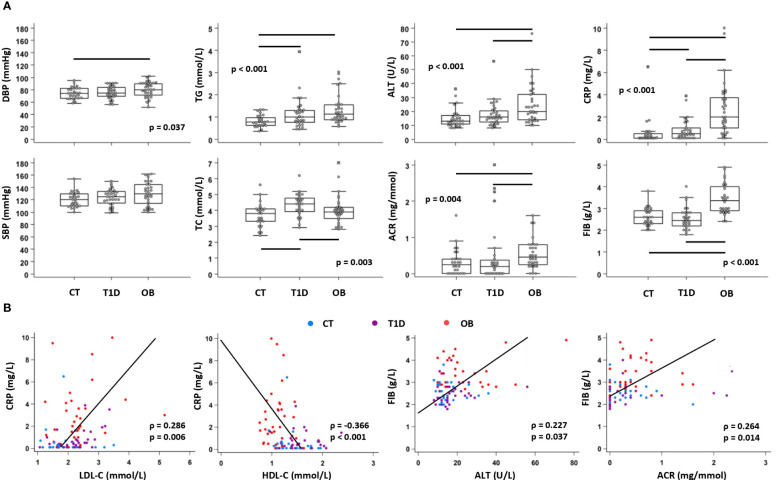
Clinical and biochemical parameters related to cardiovascular, metabolic and inflammatory status in patients with type 1 diabetes (T1D), obese (OB) patients and healthy control (CT) participants. Blood pressure was measured, and plasma biochemical parameters were determined from subjects subdivided into three groups. **(A)** Comparison of cardiometabolic risk factors between CT, T1D, and OB subjects. Individual values are presented as dots, and outlying values are presented as squares; horizontal lines represent the median, boxes represent the interquartile range (IQR), and whiskers represent 1.5 times the IQR. Statistically significant differences were determined at p <0.05, Kruskal–Wallis test followed by Conover test for further group-to-group comparisons (lines denote significant differences between groups). One outlying value for ALT and two outlying values for ACR are not shown on the graphs for clarity reasons but are included in the statistical analysis. **(B)** Association of inflammatory parameters with parameters related to metabolic syndrome including all subjects (groups are marked by different colors). Individual values and trend lines are presented with Spearman’s rank correlation coefficient (ρ). Statistically significant difference was determined at p <0.05. SBP, systolic blood pressure; DBP, diastolic blood pressure; TC, total cholesterol; LDL-C, low-density lipoprotein cholesterol: HDL-C, high-density lipoprotein cholesterol; TG, triglycerides; ALT, alanine transaminase; ACR, albumin-to-creatinine ratio in urine; FIB, fibrinogen; CRP, C-reactive protein.

Clinical laboratory parameters related to cardiovascular (DBP, SBP), metabolic (TC, HDL-C, LDL-C, TG, ALT, AST, ACR) and inflammatory (FIB, CRP) status were recorded for all three groups to assess the risk of cardiovascular and metabolic complications (**Supplementary Table 1**). Overall, cardiometabolic risk factors were higher in the OB group than in the other two groups, including DBP, ALT and ACR. TG concentration was higher in both experimental groups (OB and T1D) compared to the controls, whereas TC was higher in T1D than in the other two groups (**Figure 2A**). The lipid profile also showed higher LDL-C in both OB (2.2 (1.9–2.4) mmol/L) and T1D (2.3 (1.9–2.6) mmol/L) groups compared to the controls (1.9 (1.6–2.2) mmol/L; p=0.023), and lower HDL-C in the OB group (1.1 (0.9–1.2) mmol/L) than in the other two groups (1.3 (1.2–1.6) mmol/L in controls; 1.5 (1.3–1.8) mmol/L in T1D; p<0.001). In OB, LDL-C was positively associated with ALT (ρ=0.362, p=0.039), and TG were positively associated with HOMA-IR (ρ=0.561, p<0.001) and ALT (ρ=0.569, p<0.001). In T1D, LDL-C was positively associated with CRP (ρ=0.460, p=0.011), and TG were positively associated with HbA1c (ρ=0.543, p=0.002). Inflammatory parameters (FIB, CRP) were elevated in the OB group compared to the other two groups, whereas only CRP was elevated in the T1D group compared to the control group (**Figure 2A**). Inflammatory markers showed significant associations with parameters related to the metabolic syndrome, including CRP with LDL-C and HDL-C, and FIB with ALT and ACR (**Figure 2B**). These results confirmed that inflammation may contribute to metabolic dysfunction in T1D and OB; therefore, we further analyzed immune cell subsets and chemokine axes in the study subjects.

### 3.2 Peripheral blood immune cell distribution and plasma chemokine levels in T1D and OB

**Figure 3:**
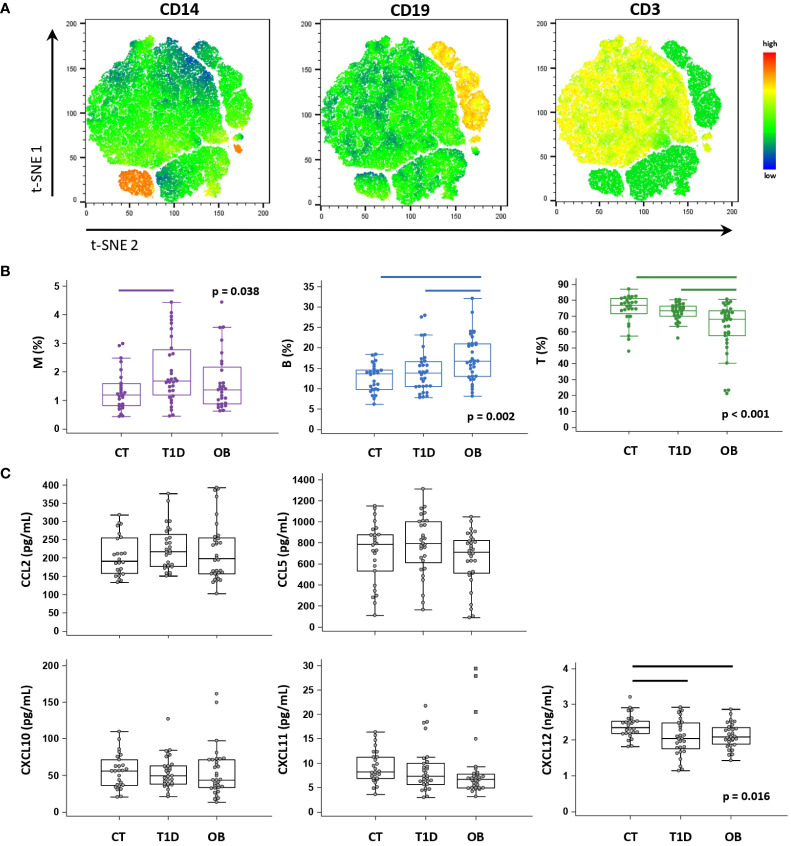
Peripheral blood immune cell distribution and plasma chemokine levels determined in patients with type 1 diabetes (T1D), obese (OB) patients and healthy control (CT) participants. Peripheral blood mononuclear cells (PBMCs) were isolated and phenotyped using flow cytometry. **(A)** Representative plots for major immune subsets within PBMCs showing CD14^+^ monocytes (M), CD19^+^ B lymphocytes (B) and CD3^+^ T lymphocytes (T). Visual presentations of cell clusters were performed by a T-distributed stochastic neighbor embedding (tSNE) algorithm using compensated fluorescence parameters for each marker (heatmap view of fluorescence intensity). **(B)** Frequencies of M, B and T subsets among total CD45^+^ cells between the three groups. **(C)** Chemokine levels detected in plasma samples using cytometric bead-based immunoassay (CCL2, CCL5, CXCL10 and CXCL11) and ELISA (CXCL12). **(B, C)** Individual values are presented as dots, and outlying values are presented as squares; horizontal lines represent the median, boxes represent the interquartile range (IQR), and whiskers represent 1.5 times the IQR. Statistically significant differences were determined at p <0.05, Kruskal–Wallis test followed by Conover test for further group-to-group comparisons (lines denote significant differences between groups).

PBL immune cell subsets were identified based on the expression of CD3 (T lymphocytes), CD19 (B lymphocytes) and CD14 (monocytes) by flow cytometry (**Figure 3A**). T1D patients had a significantly higher proportion of CD14^+^ monocytes, whereas OB patients had a significantly higher proportion of CD19^+^ B lymphocytes and a significantly lower proportion of CD3^+^ T lymphocytes (**Figure 3B**).

Among selected CC-chemokine ligands (CCL2 and CCL5) and CXC-chemokine ligands (CXCL10, CXCL11, CXCL12), we observed significantly lower CXCL12 concentrations in the T1D and OB groups than in the controls (**Figure 3C**). We further identified subgroups of T1D and OB patients at higher risk of complications (expecting them to have more pronounced immune abnormalities), based on the criteria for unsatisfactory glycemic control according to HbA1c and for severe obesity translated to BMI Z score, respectively (36, 38). T1D patients with HbA1c >8% (>64 mmol/mol) had significantly higher CCL2 concentrations (241.0 (189.6–295.3) pg/mL) than controls (191.5 (158.0–254.7) pg/mL, p=0.033), whereas OB patients with BMI Z score >2 SD had significantly lower CXCL11 concentrations (6.6 (4.9–7.7) pg/mL) than controls (8.2 (6.9–11.3) pg/mL, p=0.018) (graphs not shown).

**Figure 4:**
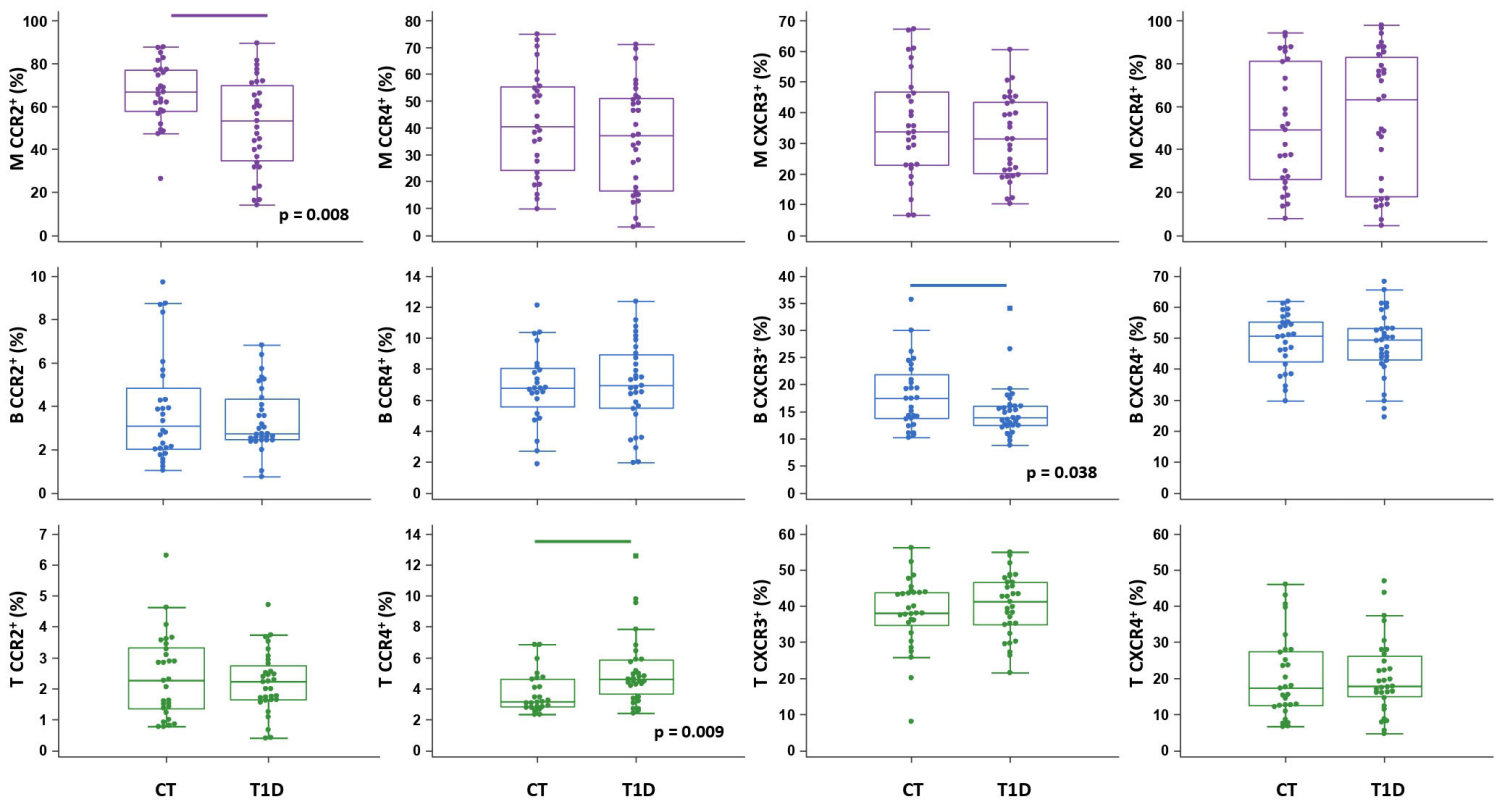
Chemokine receptor profiling of immune cells in type 1 diabetes (T1D) patients. Peripheral blood mononuclear cells (PBMCs) were isolated and phenotyped using flow cytometry. Frequencies of CD14^+^ monocytes (M), CD19^+^ B lymphocytes (B), and CD3^+^ T lymphocytes (T) expressing chemokine receptors CCR2, CCR4, CXCR3 and CXCR4 are shown as proportions of M, B and T subsets, and compared to the control group (CT). Individual values are presented as dots, and outlying values are presented as squares; horizontal lines represent the median, boxes represent the interquartile range (IQR), and whiskers represent 1.5 times the IQR. Statistically significant differences were determined at p <0.05, Mann–Whitney test (lines denote significant differences between groups).

**Figure 5:**
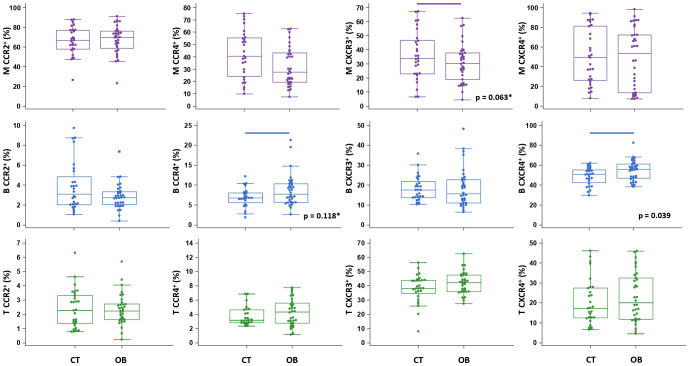
Chemokine receptor profiling of immune cells in obese (OB) patients. Peripheral blood mononuclear cells (PBMCs) were isolated and phenotyped using flow cytometry. Frequencies of CD14^+^ monocytes (M), CD19^+^ B lymphocytes (B) and CD3^+^ T lymphocytes (T) expressing chemokine receptors CCR2, CCR4, CXCR3 and CXCR4 are shown as proportions of M, B and T subsets, and compared to the control group (CT). Individual values are presented as dots, and outlying values are presented as squares; horizontal lines represent the median, boxes represent the interquartile range (IQR), and whiskers represent 1.5 times the IQR. Statistically significant differences were determined at p <0.05, Mann–Whitney test (lines denote significant differences between groups). Two comparisons with marginal significance are marked by asterisk (*).

We concluded that those chemokines may participate in the disease pathogenesis and, therefore, assessed the profile of selected corresponding chemokine receptors on PBL immune cell subsets (CCR2 and CCR4 receptors for CCL2 and CCL5; CXCR3 receptor for CXCL10 and CXCL11; CXCR4 receptor for CXCL12) (gating strategy to evaluate chemokine receptor expression on PBL immune cell subsets is shown in **Supplementary Figure 1**). In general, CD14^+^ monocytes expressed both CC- and CXC-chemokine receptors in high proportions, whereas CD19^+^ B lymphocytes and CD3^+^ T lymphocytes dominantly expressed CXC-receptors (**Figures 4**, **5**). Our further aim was to identify the disease-specific chemokine receptor profile as well as the associations of chemokine receptor expression and chemokine levels with cardiometabolic risk factors in each experimental group (**Figure 1**).

### 3.3 T1D-specific profile and association with cardiometabolic risk factors

The total proportion of CD14^+^ monocytes was higher in T1D patients and inversely associated with AST (ρ=-0.431, p=0.015) (graph not shown). CD14^+^ monocytes exhibited significantly lower expression of CCR2 in T1D patients than in controls (**Figure 4**), which was paralleled by significantly elevated levels of CCL2 in T1D patients with HbA1c >8% (>64 mmol/mol) (graph not shown). In addition, in T1D group we found significantly lower expression of CXCR3 on CD19^+^ B lymphocytes and significantly higher expression of CCR4 on CD3^+^ T lymphocytes (**Figure 4**). The total proportion of CD3^+^ T lymphocytes was positively associated with HbA1c (ρ=0.383, p=0.040) (graph not shown).

**Figure 6:**
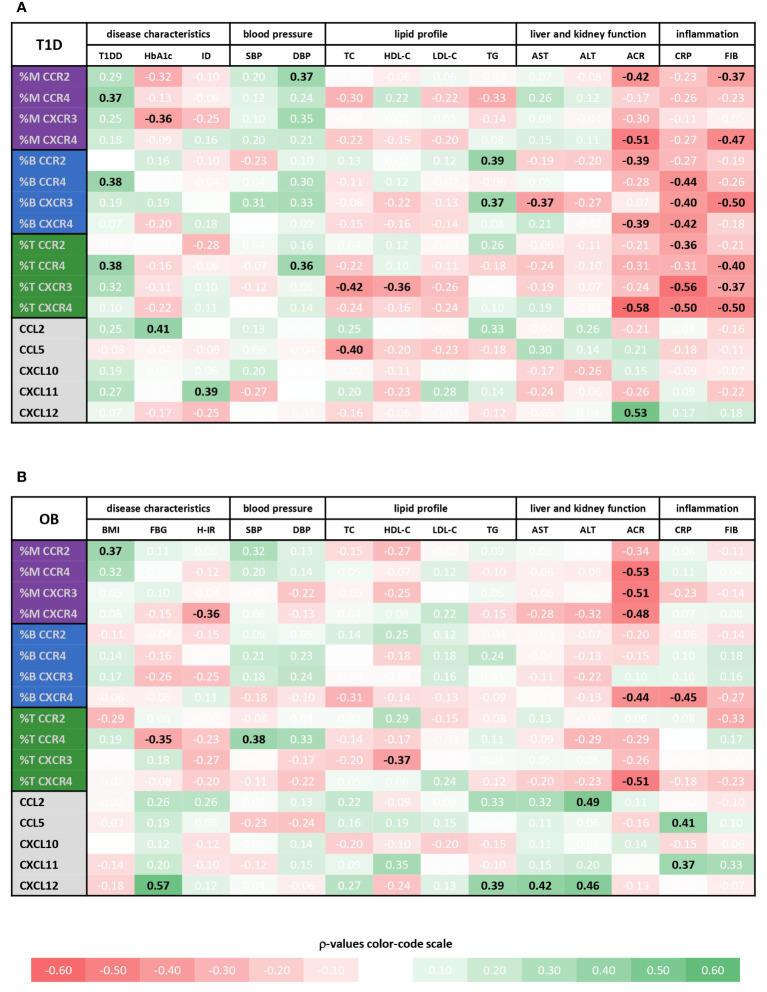
Associations of immune cell subsets expressing specific chemokine receptors and chemokine levels with cardiometabolic status of type 1 diabetes (T1D) and obese (OB) patients. Peripheral blood mononuclear cells (PBMCs) were isolated and phenotyped using flow cytometry. Chemokine levels were detected in plasma samples using cytometric bead-based immunoassay (CCL2, CCL5, CXCL10 and CXCL11) and ELISA (CXCL12). Tables show correlations of CD14^+^ monocytes (M), CD19^+^ B lymphocytes (B) and CD3^+^ T lymphocytes (T) expressing chemokine receptors (CCR2, CCR4, CXCR3 and CXCR4) as well as chemokine levels with clinical and biochemical parameters related to cardiovascular, metabolic, and inflammatory status for **(A)** T1D group and **(B)** OB group. Individual values represent Spearman’s rank correlation coefficient (ρ), which is color-coded; the red spectrum represents negative associations, and the green spectrum represents positive associations. Statistically significant differences were determined at p <0.05 and highlighted in the table as bold black numbers. T1DD, T1D duration; ID, insulin dose; BMI, body mass index Z score; FBG, fasting blood glucose; H-IR, homeostatic model assessment for insulin resistance; SBP, systolic blood pressure; DBP, diastolic blood pressure; TC, total cholesterol; LDL-C, low-density lipoprotein cholesterol; HDL-C, high-density lipoprotein cholesterol; TG, triglycerides; AST, aspartate transaminase; ALT, alanine transaminase; ACR, albumin-to-creatinine ratio in urine; CRP, C-reactive protein; FIB, fibrinogen.

Chemokine-receptor profiling in T1D group was marked by a positive association between CCR2^+^ monocytes and CCR4^+^ T lymphocytes with DBP, as well as between CCR2^+^ and CXCR3^+^ B lymphocytes with TG (**Figure 6A**), linking endothelial inflammation, atherosclerosis and dyslipidemia. CCR4^+^ subsets were positively associated with T1DD. Negative associations were found for CXCR3^+^ monocytes with HbA1c, for CXCR3^+^ B lymphocytes with AST and for CXCR3^+^ T lymphocytes with TC and HDL-C. CCR2^+^ and CXCR4^+^ subsets were negatively associated with ACR. Finally, striking negative associations were found for most subsets with CRP and FIB, which could indicate tissue recruitment under inflammatory conditions.

In contrast to well associated expression of chemokine receptors with disease parameters, only a few significant positive associations were observed for the levels of chemokine ligands, including CCL2 with HbA1c, CXCL11 with ID and CXCL12 with ACR (**Figure 6A**, **Supplementary Figure 2**).

### 3.4 OB-specific profile and association with cardiometabolic risk factors

The total proportion of CD19^+^ B lymphocytes was higher in OB patients and significantly associated with FBG (ρ=0.485, p=0.004) (graph not shown). CD19^+^ B lymphocytes also exhibited significantly higher expression of CXCR4 in OB patients than in controls (**Figure 5**), which was paralleled by significantly lower levels of CXCL12 in OB patients (**Figure 3**). In addition, CD19^+^ B lymphocyte expression of CCR4 was marginally elevated in OB patients compared to controls (p=0.118) (**Figure 5**). The expression of other chemokine receptors was similar in OB and controls, except for a marginally lower expression of CCR4 on CD14^+^ monocytes (p=0.063) (**Figure 5**).

Chemokine-receptor profiling in OB group yielded only a few positive associations, including CCR2^+^ monocytes with BMI Z score and CCR4^+^ T lymphocytes with SBP (**Figure 6B**). The most obvious significant inverse associations were found for CXCR4^+^ subsets and ACR. In addition, other monocyte subsets were inversely associated with ACR. CXCR4^+^ monocytes, CCR4^+^ T lymphocytes and CXCR4^+^ B lymphocytes were negatively associated with HOMA-IR, FBG and CRP, respectively, suggesting the role in adipose tissue inflammation. As in T1D group, CXCR3^+^ T lymphocytes were inversely associated with HDL-C.

In contrast to mainly negative associations of chemokine receptor expression with disease parameters, mostly positive associations were observed for the levels of chemokine ligands, including associations of CCL5 and CXCL11 with inflammatory markers (especially CRP), CCL2 and CXCL12 with TG, AST and ALT, and CXCL12 with FBG (**Figure 6B** and **Supplementary Figure 2**), linking inflammation with metabolic syndrome.

## 4 Discussion

Our study focused on the immune mediators underlying T1D and OB, since chronic sustained inflammation has been linked to metabolic disturbances and vascular complications in both diseases (15, 39–42). The proportions of basic PBL immune subsets differ between T1D and OB patients, suggesting a distinct immuno-pathophysiology. A significantly enlarged CD14^+^ monocyte population may contribute to inflammation associated with T1D, whereas humoral immunity with expanded CD19^+^ B lymphocytes may have a more important role in OB. These subsets were significantly associated with AST in T1D, and with FBG in OB. Although different findings on blood leukocytes have been reported in these diseases, several studies have found increased proportions of monocytes in T1D and B lymphocytes in OB (42–44).

The inflammatory marker CRP was elevated in both T1D and OB, with a positive association with LDL-C and a negative association with HDL-C. Increased CRP levels were previously reported in diabetic children, especially those with microvascular complications (21, 45), as was the positive association of CRP with BMI, LDL-C and T1DD (39). Increased CRP was also observed in overweight children, with a positive association with cardiometabolic risk factors and BMI, suggesting its usefulness as an indicator of cardiovascular complications (46, 47). Another inflammatory marker, FIB, which we found elevated in OB, was previously associated with BDP, ALT and ACR (48, 49). Parallel FIB elevation and albuminuria were observed in diabetic nephropathy and peripheral vascular disease, proposing its role in endothelial dysfunction (50).

The pathophysiology of vascular complications comprises combined effects of inflammation, endothelial dysfunction and metabolic abnormalities (46, 51, 52). We detected increased LDL-C and TG in T1D and OB groups as well as lower HDL-C in OB group, and such atherogenic patterns were commonly found in these diseases (13, 53). Our data indicate that dyslipidemia is associated with insulin resistance and liver steatosis in OB, and with inflammation and poor glycemic control in T1D. Overall, OB children had more pronounced disturbances in cardiometabolic parameters than T1D children, suggesting that even in the pediatric population, overweight dramatically impacts metabolic processes. Namely, DPB, TG, LDL-C, ALT and ACR were significantly increased in OB patients together with HOMA-IR, INS and BMI, whereas T1D patients mainly exhibited dyslipidemia (increased TC, LDL-C and TG). A higher prevalence of cardiovascular risk factors was proven for pediatric T2D compared with T1D, explaining earlier cardiovascular disease in OB children, especially in those with insulin resistance, metabolic syndrome, MAFLD and microalbuminuria (13, 54).

Since the complex network of chemokines mediates effector immune cell infiltration to target tissues (3), we performed profiling of selected chemokine ligands and receptors to reveal their associations with disease severity and cardiometabolic risk factors. However, we did not find an increase in chemokine levels, except for CCL2 in poorly controlled T1D. Moreover, CXCL11 and CXCL12 were lower in OB patients, and CXCL12 in T1D. It was previously shown that the production of chemokines may not be elevated in long-lasting inflammatory conditions and that circulatory levels may not adequately reflect their local production in visceral adipose tissue, pancreas and other affected sites (3, 4, 27, 28). In addition, cell phenotyping did not reveal striking differences in chemokine receptor profiles between groups. Expression of chemokine receptors is complexly regulated, especially in inflammation. Frequency of receptor-expressing cells depends on enhanced tissue recruitment of those cells, presence of decoy or atypical receptors, receptor downregulation to increase chemokine bioavailability or ligand/receptor complex internalization (with further degradation or recycling) (22, 27, 54, 55). Thus, in data interpretation, we focused more on the observed associations between chemokines/receptors and clinical data, expecting that they can be used as indicators of vascular complications.

A specific receptor for CXCL12, CXCR4 was expressed on a higher proportion of B lymphocytes in OB patients, whereas CXCL12 plasma concentrations were lower in OB and T1D groups compared to controls, suggesting the involvement of CXCL12/CXCR4 axis in these diseases. Several studies described a protective role of CXCL12 by showing its negative effect on angiogenesis in adipose tissue (20, 56) and regenerative properties in pancreatic β-cell survival (57, 58). Other studies, however, proposed a detrimental role of CXCL12 in adipose tissue inflammation and insulin resistance as well as in microvascular diabetic complications (19, 30). Moreover, CXCL12 may contribute to β-cell functional decline during T1D progression, through the stimulation of cytotoxic exhausted-like CD8^+^ T lymphocytes (59). In agreement with these findings, we observed positive associations for CXCL12 levels with FBG, TG, AST and ALT in OB group, but only with ACR in T1D group. Liu et al. found serum CXCL12 to positively correlate with BMI, SBP, ALT, AST, HOMA-IR, TC, TG and LDL-C, as well as MAFLD in OB children (60). In addition, our study revealed inverse associations of CXCR4^+^ subsets with ACR and inflammatory markers in OB group and, even more strikingly, in T1D group. These results may point to a different background of albuminuria, being a microvascular complication of hyperglycemia in T1D children, but a consequence of the metabolic syndrome in non-diabetic OB children.

The chemokines CXCL10 and CXCL11 may both bind to the CXCR3 receptor, which is expressed by a lower proportion of B lymphocytes in T1D. Smaller proportions of T and B lymphocytes expressing CXCR3 were observed in young adults with long-lasting T1D (27, 61). The proportion of CXCR3^+^ B lymphocytes was inversely associated with liver enzymes and inflammatory markers in T1D but not in OB. Recent study by Reijm et al. proposed that CXCR3^+^ activated B lymphocytes may represent an autoreactive subset important for the pathogenesis of arthritis as well as other autoimmune diseases (62). The mainly negative associations between the proportion of CXCR3^+^ T lymphocytes and cardiometabolic parameters in T1D and OB may be explained by the reported role of the CXCR3 receptor in T lymphocyte migration to the inflamed visceral adipose tissue (63). Moreover, due to its scavenger function, reduced CXCR3 expression may lead to increased ligand bioavailability (27). Levels of CXCL11 were positively associated with ID in T1D and CRP in OB. Elevated levels of CXCL10 and CXCL11 in new-onset T1D, and a decrease in CXCL10 level with disease duration were previously reported (3, 27). CXCL10 and its receptor CXCR3 are expressed in the pancreatic β-cell microenvironment in early T1D (64), so CXCL10/CXCR3 antagonists may potentially postpone T1D development (3). Severely OB adults had elevated circulating levels of CXCL10 and CXCL11, which positively correlated with waist circumference, BMI and HOMA-IR (29). Kochumon et al. reported higher adipose tissue expression of those chemokines and the association of adipose tissue-derived CXCL11 with cardiovascular risk factors (CRP, FBG, and HOMA-IR) in nondiabetic OB adults (28).

Signature monocyte attractants, CCL2 and CCL5, bind to CCR2^+^ and CCR4^+^ monocytes, but small subsets of T and B lymphocytes express those receptors as well. A lower proportion of CCR2^+^ monocytes and, inversely, a higher level of CCL2 in T1D patients with poor glycemic control indicate possible cell recruitment to the sites of tissue damage and inflammation. This is additionally supported by the inverse association of CCR2^+^ monocytes with ACR in both T1D and OB. In T1D, positive associations were found for CCR2^+^ monocytes and B lymphocytes with DBP and TG, respectively. The proportion of CCR4^+^ T lymphocytes was higher in T1D, with a positive association with BP in both T1D and OB. Moreover, all CCR4^+^ subsets were positively associated with T1DD, linking CCR4 expression to disease progression. Several chemokines may bind CCR4, including selective agonists CCL17 and CCL22, as well as CCL2, CCL3 and CCL5 in high concentrations (65, 66). CCR4 is expressed on Th2 lymphocytes, as opposed to CXCR3 that is expressed on Th1 lymphocytes, and has a proven role in some inflammatory diseases and hypersensitivity. CCL2 levels were positively associated with HbA1c in T1D, and with ALT in OB, in line with the studies describing the proinflammatory role of CCL2 in these diseases (3, 31, 67, 68). Positive associations of CCL2 levels with BMI, proportion of fat tissue, waist circumference and FBG were demonstrated in OB children (67–69). A recent study by Kostopoulou et al. included parallel analysis of children and adolescents (2–18 years) with T1D and OB compared to controls and found a significant positive correlation for CCL2 with BMI Z score by including all participants or only T1D (70). In contrast, no associations were found for CCL2 with HbA1c or metabolic factors in T1D (31, 71). There are reports of lower levels of CCL2 in T1D patients, whereas increased levels were associated with diabetic complications (31, 33). Finally, the positive association of CCL5 with CRP in OB group is in agreement with previous findings, indicating its role in adipose tissue inflammation (72).

To the best of our knowledge, this is the first study profiling chemokine ligands and receptors comparatively between pediatric T1D and OB patients. We are aware that our study design is burdened with certain limitations. Since our aim was to assess the chronic effects of inflammation in T1D patients without serious diabetic complications, we included children with long-lasting disease (median duration 10 years). Therefore, we missed possible initial inflammatory changes in chemokine levels as well as more pronounced inflammatory disturbances associated with chronic diabetic complications. Additional studies are required to further evaluate local and systemic chemokine activity at different stages of T1D and OB. Another shortcoming was the large variance of data for chemokine receptor expression that, in combination with a relatively modest sample size (~30 per group), prevented significant differences. Finally, by using a cross-sectional design we were not able to show causality for the role of chemokine/receptor profiles in cardiometabolic complications, which would require prospective follow-up. However, we believe that our findings point to possible candidates for further mechanistic studies and therapeutic targeting.

## 5 Conclusions

In summary, we revealed some overlapping findings for the role of chemokines in T1D and OB. Inverse association of CXCR4^+^ subsets with albuminuria may indicate the role of CXCL12 in kidney infiltration and damage. Additionally, the inverse associations of CXCR3^+^ T lymphocytes with dyslipidemia suggest their recruitment to adipose tissue by CXCL10 or CXCL11. Finally, positive associations of CCR4^+^ T lymphocytes with hypertension link this cell subset with vascular inflammation. Common association pattern in both conditions suggests CCR4, CXCR3 and CXCR4 as promising candidates for further exploration, as their blockade may prevent chemotaxis of pathogenic cells. Inhibitors of these chemokine receptors (in the form of neutralizing antibodies, modified ligands or small molecule inhibitors) have been tested in different diseases, but only a few of them showed approvable safety profiles and efficiency for clinical usage (73). The CXCR4 antagonist (Plerixafor) and anti-CCR4 monoclonal antibody (Mogamulizumab) are clinically approved for certain types of malignancies (74, 75). At the same time, they have been suggested as possible anti-inflammatory strategies, with the aim to block chemotaxis of inflammatory cells and to alleviate target tissue damage (66, 76). Several small-molecule inhibitors of CXCR3 have been tested in experimental studies, with AMG487 being clinically trialed for treatment of inflammatory and autoimmune diseases (77, 78). Christen and Kimmel suggested that combinatorial therapy that would include conventional therapeutics together with neutralization of the chemokine axis may prove beneficial due to targeting separate steps in immunopathogenesis (3). Anti-chemokine therapy may be further investigated for repurposing to treat T1D and OB pediatric patients at risk of cardiometabolic complications. However, such attempts should be considered with great caution, due to the complex and redundant network of chemokines, as well as their important role in tissue development and homeostasis.

## Data availability statement

The raw data supporting the conclusions of this article will be made available by the authors, without undue reservation.

## Ethics statement

The study was approved by the ethics committees of the School of Medicine, University of Zagreb and UHC Zagreb. The studies were conducted in accordance with the local legislation and institutional requirements, following the Declaration of Helsinki. Written informed consent for participation in this study was provided by the participants' legal guardians/next of kin.

## Author contributions

AŠU: Conceptualization, Data curation, Formal analysis, Investigation, Methodology, Writing – original draft, Writing – review & editing. MF: Conceptualization, Formal analysis, Investigation, Methodology, Visualization, Writing – original draft, Writing – review & editing. AŠ: Formal analysis, Investigation, Methodology, Writing – review & editing. TK: Formal analysis, Funding acquisition, Investigation, Methodology, Writing – review & editing. NK: Formal analysis, Investigation, Methodology, Writing – review & editing. DG: Conceptualization, Data curation, Formal analysis, Funding acquisition, Investigation, Methodology, Resources, Supervision, Validation, Visualization, Writing – original draft, Writing – review & editing.
