# Supplementary material for: Distinct association patterns of chemokine profile and cardiometabolic status in children and adolescents with type 1 diabetes and obesity
Source: Front Endocrinol (Lausanne). 2024 Jul 23;15:1335371. doi: 10.3389/fendo.2024.1335371 (PMC11300205; doi:10.3389/fendo.2024.1335371)
Supplement: Supplementary file 1 [file DataSheet_1.pdf]

Supplementary table 1. Demographic characteristics and clinical parameters related to cardiovascular, metabolic and inflammatory status of study participants (control group, children with type 1 diabetes and obese children)

Variables†	Control group (n=29)	T1D (n=31)	OB (n=34)	p‡
**Age (years)**	15.0 [14.0 – 17.0]	15.0 [14.0 – 17.0]	14.0 [14.0 – 16.3]	0.115
**Sex (female/male)**	14/15 (48.3/51.7)	19/12 (61.3/38.7)	19/15 (55.9/44.1)	0.596
**BMI (Z score)**	0.10 [-0.68 – 0.55]	-0.12 [-0.69 – 0.68]	**2.35 [2.16 – 2.52]**	**<0.001**
**SBP (mmHg)**	120.0 [110.0 – 130.0]	125.0 [115.0 – 133.5]	130.0 [114.5 – 145.0]	0.079
**DBP (mmHg)**	74.0 [66.0 – 82.0]	75.0 [69.3 – 83.8]	**80.0 [71.5 – 90.0]**	**0.037**
**TC (mmol/L)**	3.80 [3.30 – 4.10]	**4.40 [3.93 – 4.78]**	3.90 [3.50 – 4.20]	**0.003**
**LDL-C (mmol/L)**	1.89 [1.59 – 2.20]	**2.26 [1.89 – 2.60]**	**2.20 [1.94 – 2.44]**	**0.023**
**HDL-C(mmol/L)**	1.32 [1.20 – 1.59]	1.47 [1.28 – 1.76]	**1.08 [0.92 – 1.20]**	**<0.001**
**TG (mmol/L)**	0.77 [0.62 – 0.97]	**1.00 [0.76 – 1.30]**	**1.13 [0.88 – 1.55]**	**<0.001**
**HOMA-IR**	1.88 [1.57 – 2.86]	N/A	**4.32 [2.99 – 6.20]**	**<0.001**
**HbA1c (%)**	N/A	8.20 [7.43 – 9.20]	N/A	
**FBG (mmol/L)**	4.70 [4.38 – 4.80]	N/A	4.70 [4.40 – 5.00]	0.430
**INS (μIU/mL)**	9.40 [7.80 – 13.73]	N/A	**19.65 [14.90 – 27.40]**	**<0.001**
**AST (U/L)**	21.00 [15.75 – 22.25]	21.00 [18.00 – 25.00]	20.50 [18.00 – 24.00]	0.364
**ALT (U/L)**	13.00 [11.00 – 17.00]	16.00 [12.25 – 20.50]	**21.50 [14.00 – 33.00]**	**<0.001**
**AST/ALT< 1**	3 (10.3%)	5 (16.1%)	**16 (47.1%)**	**0.001**
**ACR (mg/mmol)**	0.25 [0.00 – 0.40]	0.20 [0.00 – 0.38]	**0.50 [0.28 – 0.80]**	**0.004**
**FIB (g/L)**	2.60 [2.30 – 2.90]	2.45 [2.20 – 2.80]	**3.40 [2.90 – 4.08]**	**<0.001**
**CRP (mg/L)**	0.1 [0.1 – 0.5]	**0.5 [0.1 – 1.0]**	**2.2 [1.0 – 4.1]**	**<0.001**

† T1D – children affected by type 1 diabetes for more than five years; OB – children with BMI above the 95th percentile; BMI – body mass index (Z score); SBP – systolic blood pressure; DBP – diastolic blood pressure; TC – total cholesterol; LDL-C – low-density lipoprotein cholesterol: HDL – high-density lipoprotein cholesterol; TG – triglycerides; HOMA-IR – homeostatic model assessment for insulin resistance; FBG – fasting blood glucose; INS – insulin; AST – aspartate transaminase; ALT – alanine transaminase; ACR – albumin-to-creatinine ratio in urine; FIB – fibrinogen; CRP – C-reactive protein; N/A – not applicable. Values are presented as median with the interquartile range, except AST/ALT ratio and sex categories that are presented as absolute numbers and percentage of total.

‡ Bolded values – significantly different from the control group according to: Kruskal-Wallis test followed by Conover test for group-to-group comparisons; or Chi-square test for categorical values. Statistically significant difference was determined at p <0.05.

Supplementary table 2. Details of monoclonal antibodies used for flow cytometry analysis

**Marker**	**Clone**	**Fluorochrome**	**Manufacturer**	**Catalog number**
**CD3**	OKT3	Alexa Fluor® 488	BioLegend (San Diego, CA, USA)	317310
**CD14**	63D3	PE/Cyanine7	BioLegend (San Diego, CA, USA)	367112
**CD19**	SJ25C1	APC/Fire™ 750	BioLegend (San Diego, CA, USA)	363030
**CD45**	HI30	PerCP	BioLegend (San Diego, CA, USA)	304026
**CCR2 (CD192)**	48607	PE	R&D Systems (Bio-Techne, Abingdon, UK)	FAB151P
**CCR4 (CD194)**	D8SEE	APC	eBiosciences (San Diego, CA, USA)	17-1949-42
**CXCR3 (CD183)**	G025H7	APC	BioLegend (San Diego, CA, USA)	353708
**CXCR4 (CD184)**	1265	PE	BioLegend (San Diego, CA, USA)	306506

Supplementary figure 1

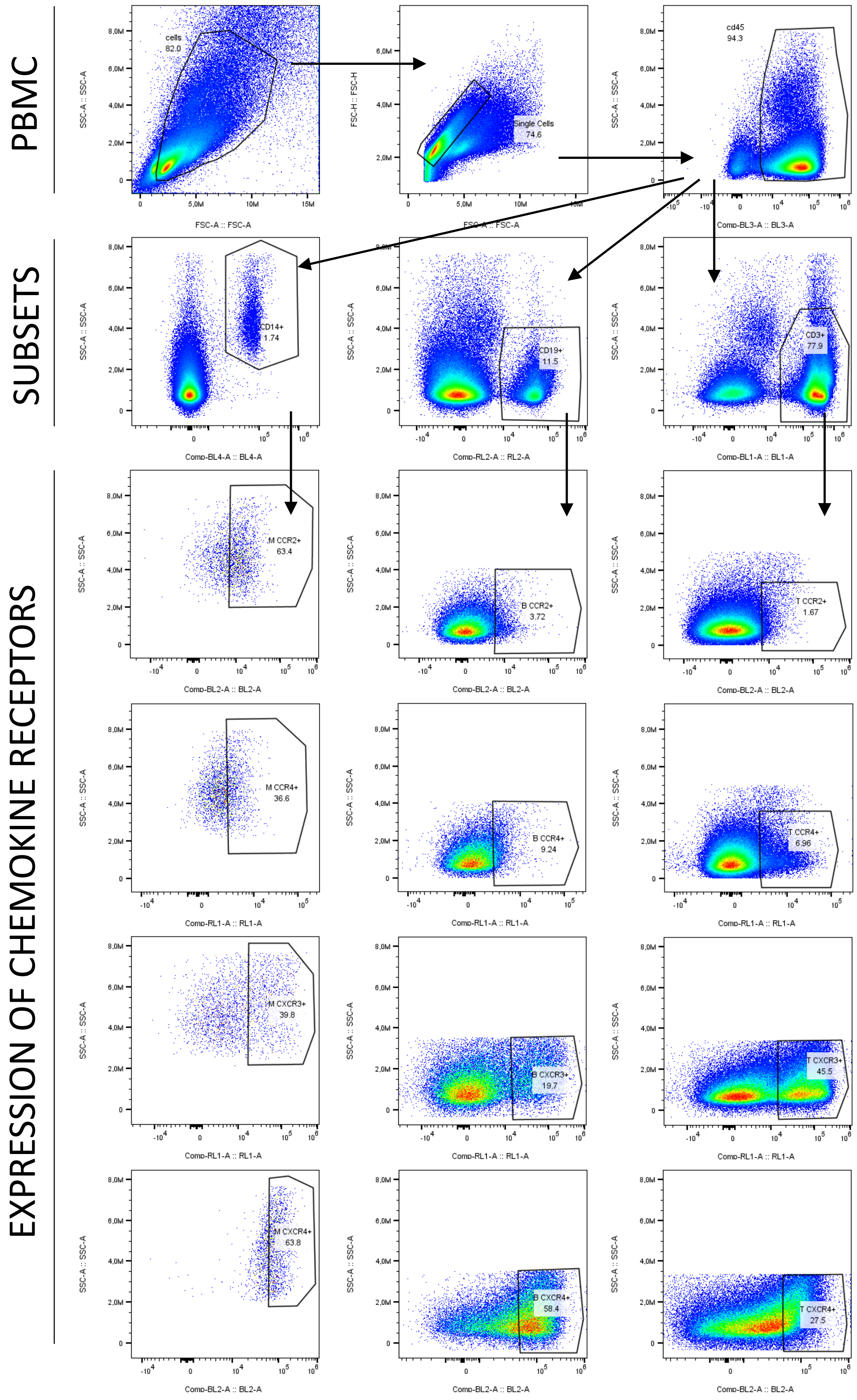

Supplementary figure 1. Gating strategy to evaluate chemokine receptor expression on peripheral blood immune cell subsets. Peripheral blood mononuclear cells (PBMCs) were isolated by density separation. Applied gating strategy first included delineation of hematopoietic ($CD45^+$) singlets, followed by gating of immune cell subsets: monocytes ($CD14^+$), B lymphocytes ($CD19^+$) and T lymphocytes ($CD3^+$), which were further assessed for chemokine receptor expression (CCR2, CCR4, CXCR3, CXCR4).

Supplementary figure 2

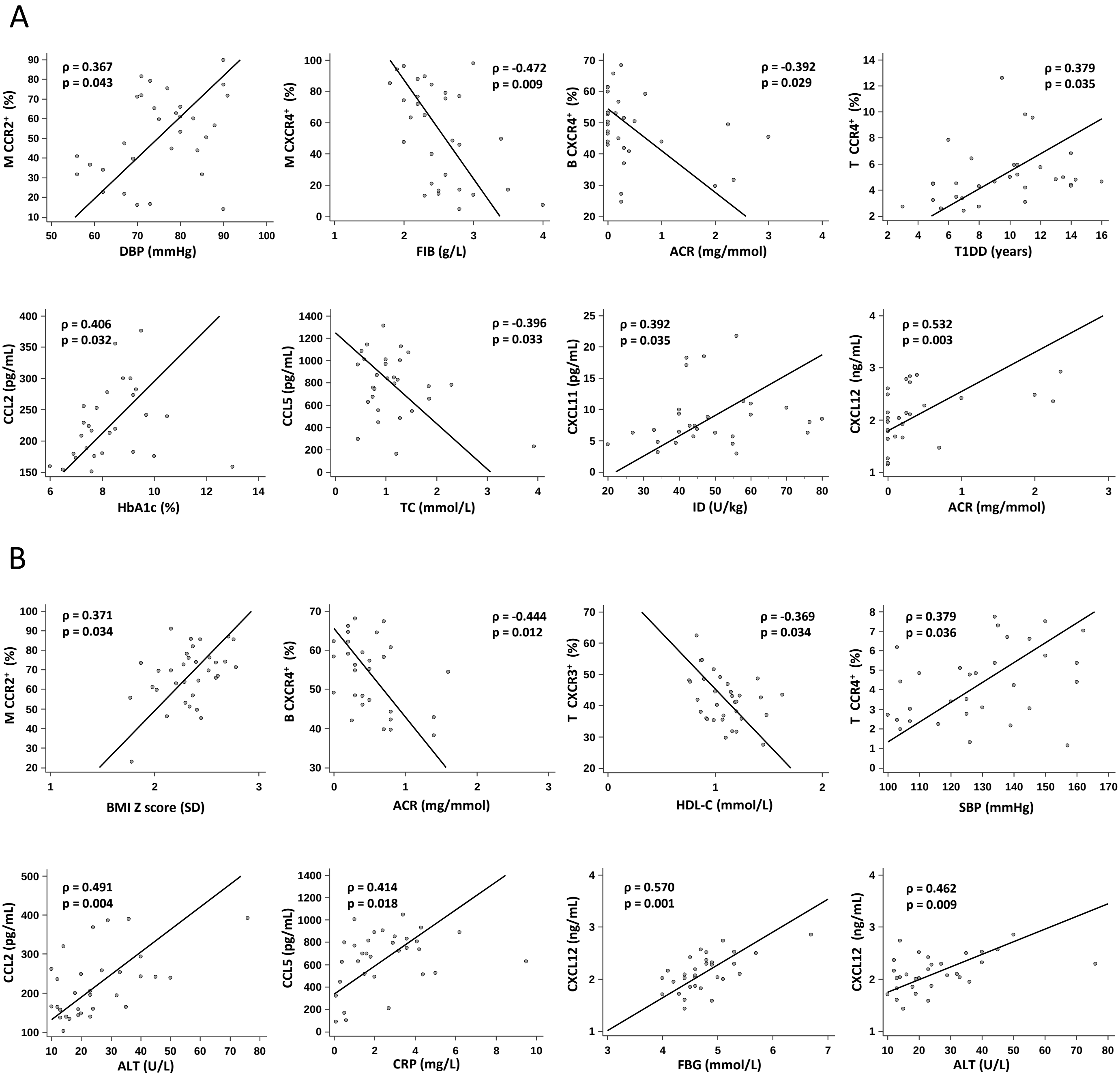

Supplementary figure 2. Association of chemokine level and receptor expression with inflammatory and cardiometabolic parameters. Selected significant positive and negative associations for: (**A**) type 1 diabetic and (**B**) obese patients. Individual values and trend lines are presented with the Spearman's rank correlation coefficient (ρ). Statistically significant difference was determined at $p < 0.05$. M – monocytes; B – B lymphocytes; T – T lymphocytes; SBP – systolic blood pressure; DBP – diastolic blood pressure; TC – total cholesterol; HDL-C – high-density lipoprotein cholesterol; TG – triglycerides; ALT – alanine transaminase; ACR – albumin-to-creatinine ratio in urine; FIB – fibrinogen; CRP – C-reactive protein; T1DD – type 1 diabetes duration; HbA1c – glycated hemoglobin; ID – insulin dose; BMI – body mass indeks Z score; FBG – fasting blood glucose.
